# Binding Sites Analyser (BiSA): Software for Genomic Binding Sites Archiving and Overlap Analysis

**DOI:** 10.1371/journal.pone.0087301

**Published:** 2014-02-12

**Authors:** Matloob Khushi, Christopher Liddle, Christine L. Clarke, J. Dinny Graham

**Affiliations:** 1 Westmead Institute for Cancer Research, Sydney Medical School, University of Sydney and the Westmead Millennium Institute, Westmead, New South Wales, Australia; 2 Storr Liver Unit, Sydney Medical School, University of Sydney and the Westmead Millennium Institute, Westmead, New South Wales, Australia; 3 Australian Breast Cancer Tissue Bank, Sydney Medical School, University of Sydney and the Westmead Millennium Institute, Westmead, New South Wales, Australia; University of Rome Tor Vergata, Italy

## Abstract

Genome-wide mapping of transcription factor binding and histone modification reveals complex patterns of interactions. Identifying overlaps in binding patterns by different factors is a major objective of genomic studies, but existing methods to archive large numbers of datasets in a personalised database lack sophistication and utility. Therefore we have developed transcription factor DNA binding site analyser software (BiSA), for archiving of binding regions and easy identification of overlap with or proximity to other regions of interest. Analysis results can be restricted by chromosome or base pair overlap between regions or maximum distance between binding peaks. BiSA is capable of reporting overlapping regions that share common base pairs; regions that are nearby; regions that are not overlapping; and average region sizes. BiSA can identify genes located near binding regions of interest, genomic features near a gene or locus of interest and statistical significance of overlapping regions can also be reported. Overlapping results can be visualized as Venn diagrams. A major strength of BiSA is that it is supported by a comprehensive database of publicly available transcription factor binding sites and histone modifications, which can be directly compared to user data. The documentation and source code are available on http://bisa.sourceforge.net

## Introduction

The recent revolution in whole genome census approaches has seen an exponential increase in available data sets describing genomic features, such as transcription factor (TF) binding sites and histone modifications. Recent studies have revealed that there are often overlaps and co-association between transcription factors at binding sites [1], [2] and identifying relationships, such as overlaps in genomic features, has become a fundamental biological research tool [3]. Moreover, the existence of a wealth of published data sets now presents unprecedented opportunities for data mining in large databases of archived genomic data.

Existing methods of finding overlaps such as BEDTools, UCSC Table Browser, Homer or Segtor [4], [5], [6] are limited in functionality for simultaneous comparison to multiple archived data sets. Moreover, few tools provide a simple interface that can be easily implemented by biologists with limited computing skills.

To address these challenges, we have developed BiSA, which is pre-loaded with transcription factor binding sites and histone modification locations, for a range of cell-types and conditions, reported in previous ChIP-chip and ChIP-Seq studies. BiSA allows the investigator to analyse overlapping or non-overlapping regions, to visualise results by Venn diagram, and to identify the genes located near to regions under study. BiSA is controlled through a user-friendly graphical user interface (GUI), installed on a Windows environment or embedded in the Galaxy web-based high throughput genomic analysis tool. Both options maximise the ease of use of this powerful tool for molecular biologists, who may lack the necessary computing skills required to use alternate approaches.

## Methods

BiSA employs a rational database management system-based architecture to archive unlimited numbers of binding datasets in a very flexible and convenient format. BiSA is developed in C# and SQL Server 2008 for the Windows environment, while Python and PostgreSQL have been used to develop a Linux version that runs under the Galaxy [7] web-based environment. We have used Google Charts to generate Venn diagrams. To calculate common sections on Venn diagrams for three datasets, BiSA first extracts overlapping regions of two datasets and then overlaps the result with the third dataset.

There are three main steps of installation of BiSA for Windows: i) installation of the MS SQL Server database engine. BiSA is fully compatible with the free Express Edition of SQL Server [8], ii) downloading and restoring the BiSA database file using SQL Server Management Studio, and iii) linking the front-end application to the database. Detailed step-by-step installation instructions with screenshots for Windows and Linux environments are available at the project website http://bisa.sourceforge.net/. We will periodically update the database to include datasets from the latest published studies.

The BiSA database schema is straightforward. All information about a dataset such as factor label, cell line and condition are saved in the ‘kbdetails’ table while the genomic region data are saved in the ‘kbsites’ table and linked to the ‘kbdetails’ table by an identity. There are four gene annotation tables covering the human hg19 and hg18, and mouse mm9 and mm8 genome assemblies. In addition to the options provided by the graphical user interface (GUI), a user can analyse data via SQL Server Management Studio by structured query language (SQL). SQL is an accessible database interrogation language, and simple SQL statements can be used to analyse data, for example:

1. Erroneous reporting of an end coordinate smaller than the start coordinate for a genomic region can be discovered by the SQL statement: SELECT * FROM kbsites WHERE [end]<start Running this query on the BiSA KB, interrogated ∼18 million regions in less than 1 second and discovered one dataset where this error was present.
2. The gene annotation tables in BiSA contain gene names and symbols, NCBI accession IDs, chromosome, strand, and the coordinates of transcription start site (TSS), end site (TES), coding sequence (CDS) and exon positions. To return all annotation details in the hg19 assembly for the breast cancer susceptibility gene BRCA1, an investigator could use the query: SELECT * FROM Annotation_hg19 WHERE gene_id = ‘BRCA1’
3. To report all genomic features within 100 kb upstream or downstream of the BRCA1 TSS. The user could then use the coordinates returned in example 2, in the SQL query: SELECT * FROM kbsites WHERE chr = ‘chr17’ AND start> = 41096311 AND start< = 41296311
4. Average region length in dataset ID 10 can be retrieved by the SQL statement: SELECT AVG([end] - start) FROM kbsites WHERE kbid = 10

Initially we have populated the BiSA database with ∼600 datasets of transcription factor binding sites and histone modifications amounting to approximately 18 million genomic regions. The data have been collected from previously published studies deposited on the publishing journals' websites, Gene Expression Omnibus (GEO), European Bioinformatics Institute (EBI), Cistrome Project [9] and some datasets are collected directly from the authors. The source of the data and additional comments, if there are any, are recorded in the kbdetails table. Addition of more datasets is a straightforward process. We refer to the sum of these datasets as the Knowledge Base (KB). Users can expand on an existing KB or build their own KB. The KB, currently, comprises human (hg18 & hg19 build) and mouse (mm8 and mm9) assemblies.

BiSA steps through the process of studying binding region interaction, annotation, statistical significance and management of datasets in seven GUI screens. An investigator can study overlapping regions by setting the minimum base pair (bp) overlap. It is also possible in BiSA to limit reported overlaps based on a maximum distance between binding peaks. This is important since binding region boundaries are highly dependent on the peak caller software and parameters used.

We have implemented IntervalStats [10] in BiSA to test the statistical significance of overlap between two dataset. IntervalStats is a command line tool written mainly for the Unix environment. Therefore, we used the MinGW toolkit [11] to compile it for the Windows environment. The BiSA for Windows download package includes an IntervalStats executable file and dependent DLL libraries, however, the tool runs independently of BiSA. When the IntervalStats tool is executed through the BiSA GUI, the datasets under study are saved in the ‘data’ subfolder and the files are passed to the IntervalStats tool. During the execution of the statistical tool the terminal window stays open to show the messages from the tool. IntervalStats calculates a p-value for each region in a query dataset against the nearest region from a reference dataset. A defined domain dataset, representing the line-space of possible interval locations, acts as a background to the statistical test and can be restricted to specific locations, such as promoter proximal regions, to take into account known biases in binding site detection. In the simplest case, the domain comprises the entire genome. We have populated BiSA with a number of domain files such as promoter regions within 10 kb of a TSS, intergenic regions and whole genome for hg19, hg18, mm9 and mm8 assemblies. Users can select one of the prepopulated domains or can specify a BED file as the domain. In addition to individual p-values for region overlap, IntervalStats returns a summary statistic, referred to as the Overlap Correlation Value, to identify the overall significance of overlap of two datasets. This summary statistic represents the fraction of regions in the query dataset with a p-value of overlap to the reference below a significance threshold value, and thus reflects the likely significance of overlap of the query and reference datasets. The correlation coefficient can range from 0 to 1, the closer the value to 1 the stronger the significance of overlap of two datasets. We have set the threshold p-value to 0.05, however this value can be changed in the configuration file, BiSA.exe.config if desired.

Gene annotations are obtained from the UCSC genome browser and will be updated periodically. Initially we have populated annotations for reference genomes hg18, hg19, mm8 and mm9. Custom gene definitions or transcription factor binding sites or epigenetic modifications for additional genomes for other organisms can be uploaded in the software.

## Results

### The BiSA Windows GUI is split across seven tabs

#### i) Import Datasets to Knowledge Base (KB)

**Figure 1:**
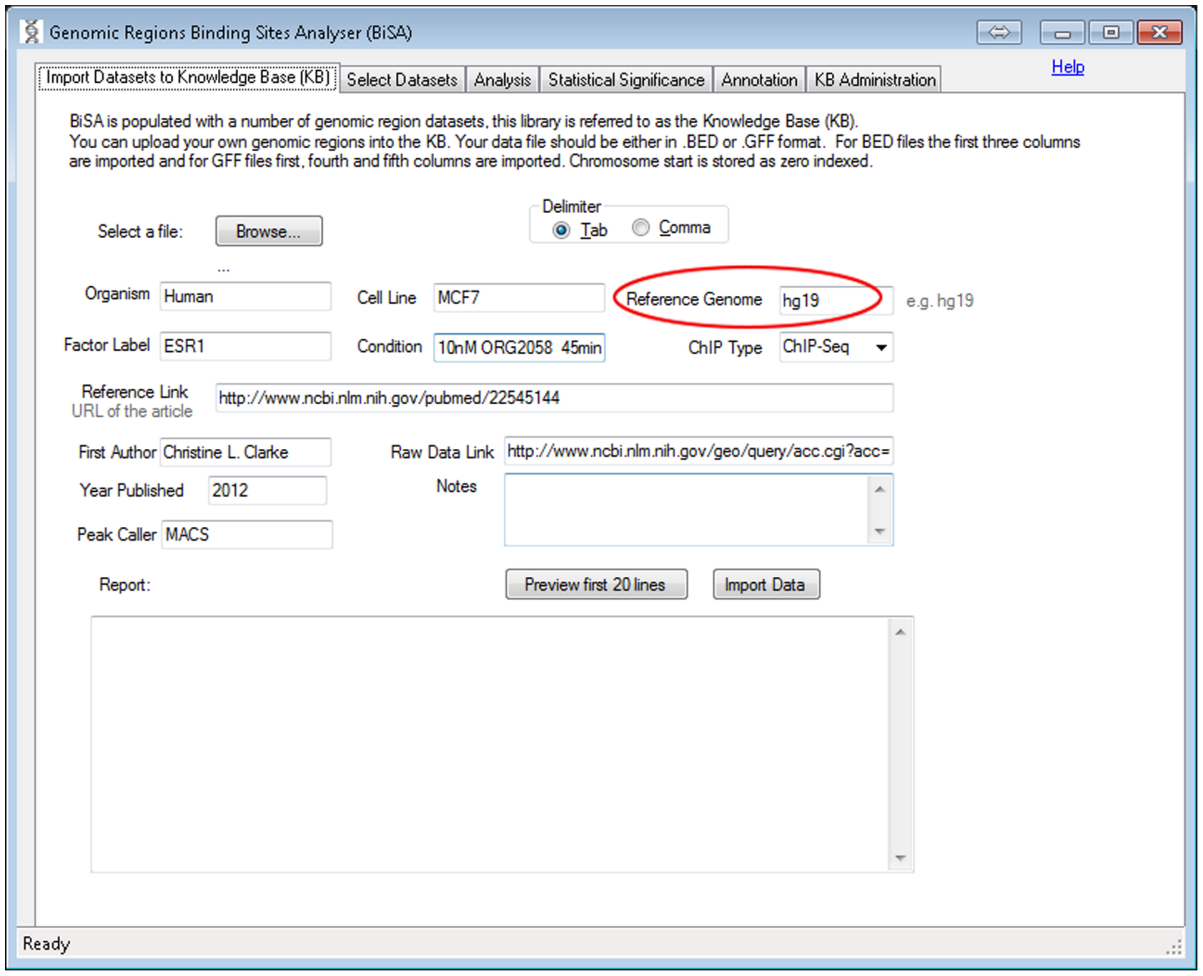
Import Datasets to Knowledge Base (KB). This step is optional and users can study data already saved in the KB, without importing datasets. In this step, the user can upload their own transcription factor DNA binding sites or histone modification locations, usually as BED or GFF peak files. If the file extension is other than BED or GFF, BiSA prompts the user to choose the right format. It is important to specify a Reference Genome (encircled), for instance hg18/hg19 for human or mm9/mm8 for mouse. BiSA will only allow comparisons between datasets of the same reference genome.

This is an optional step as the user can choose to analyse only data already contained in the KB. The user browses for their dataset, which can be uploaded in tab delimited or comma delimited BED or GFF format, assigns a logical name and description for the data, and uploads to the KB (Figure 1). The first 20 lines of the data can be displayed for verification. Chromosome position is 0 indexed as in BED format. Comments or header information in the file are reported as failed records in the ‘Report’ section (Figure 1). If no valid data are imported in the first 50 lines, the upload fails and BiSA stops the import process. The user enters information about organism and cell line, TF and conditions, which are saved with the database record. The genome build for the genomic region coordinates must be entered during this process (Figure 1, circled) and the record will be limited in future analyses to comparison with other datasets generated in the same genome and build. Associated data and publication links can also be added at this stage.

#### ii) Select Datasets

**Figure 2:**
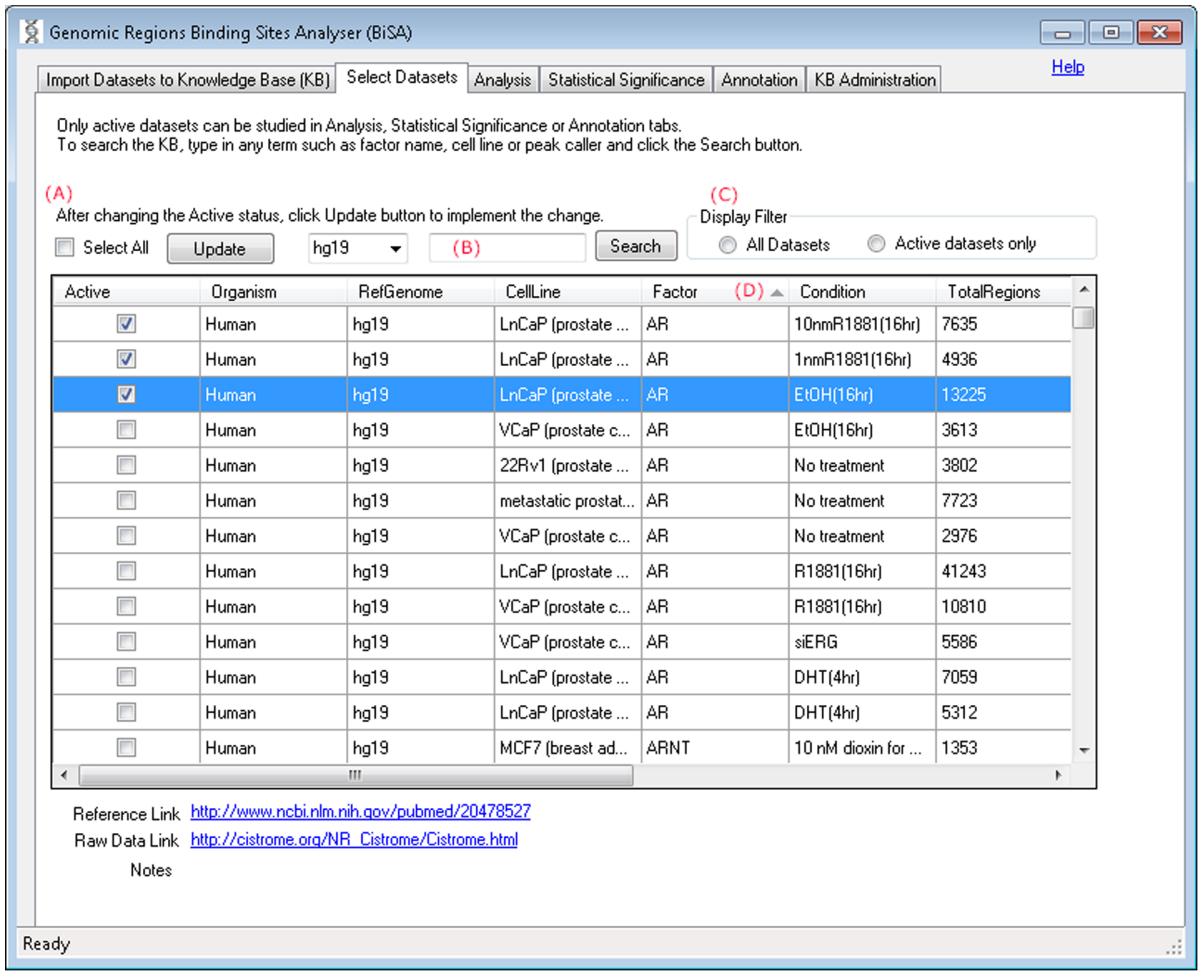
Select Datasets. This tab displays a list of all datasets in the KB, populated by default or as a consequence of uploading in Step-1. Clicking on the text of any row displays the reference link of the article, raw data link and notes, if any, below the table. Website addresses are hyperlinked to the websites/articles from where the data are obtained. (A) Changing the Active ticks and clicking on the Update button implements the selection. (B) Users can search the KB by organism, cell line, factor label, reference genome or peak caller.

This tab displays a list of all datasets in the KB including those uploaded in Step-1. Data are selected for analysis by checking the “active” box beside the relevant dataset (Figure 2A). Only data from matching reference genomes can be selected for analysis. A checked tick in the ‘Active’ column represents an active dataset that can be used in analysis in Step-3, and only active datasets can be annotated. Users can only activate datasets of matching reference genomes. To change the active status of datasets from one reference genome (e.g. hg18) to another (e.g. mm9), the user must deactivate all datasets first, which can be done by toggling on and off the ‘Select All’ check box and pressing the Update button. Clicking on the text of any row displays further information about the data. Website addresses are hyperlinked to the source websites/articles for the data. After selecting datasets for analysis, clicking on the Update button activates datasets in the BiSA database. The search field (Figure 2B) allows the user to search the KB by organism, cell line, factor label, reference genome or peak caller. Only datasets that are active can be displayed by checking the ‘Active datasets only’ option in the ‘Display Filter’ (Figure 2C). Displayed data can be sorted according to any of the database fields by selecting the column heading for the field of choice (Figure 2D).

#### iii) Analysis

**Figure 3:**
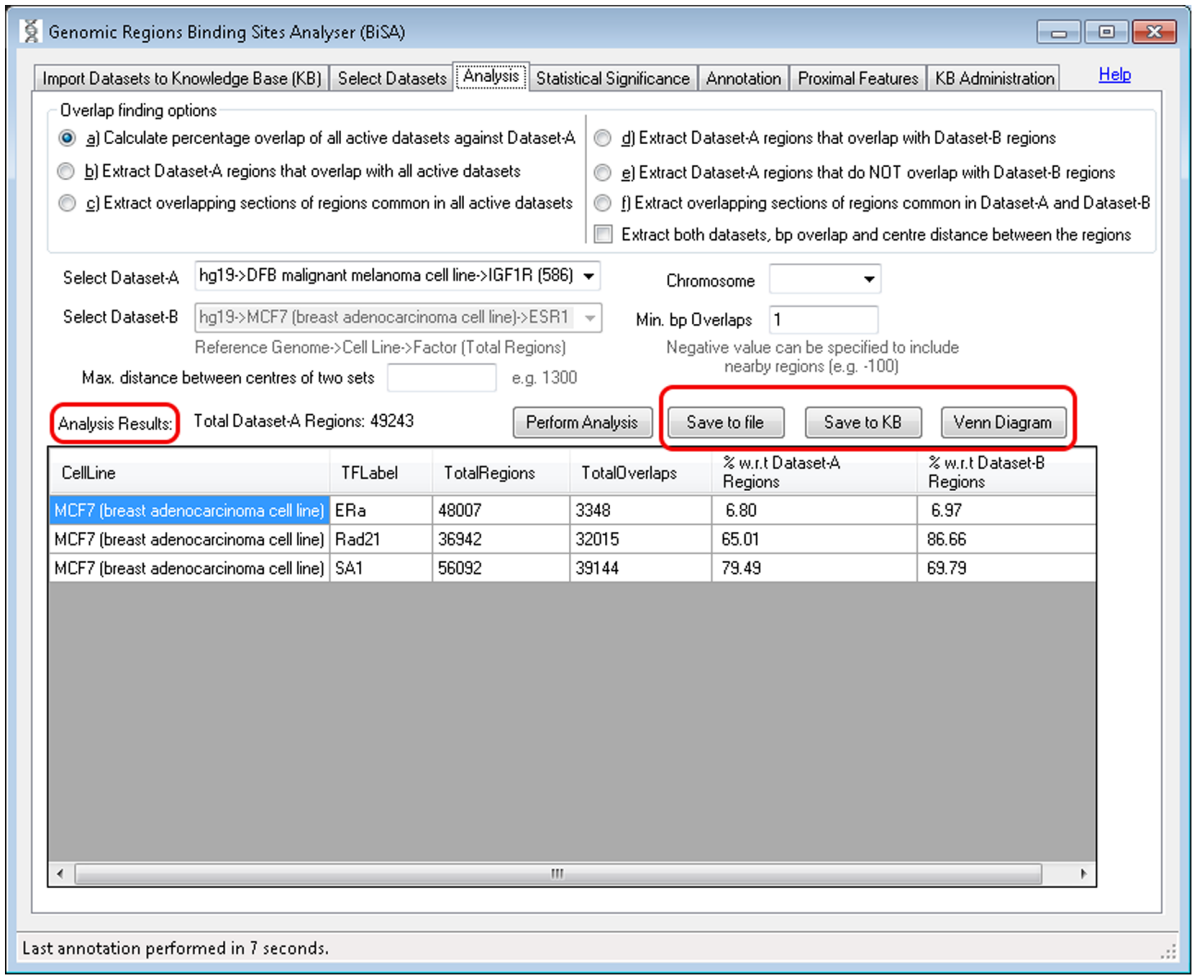
Analysis is the main overlap analysis tab of BiSA. BiSA offers six types of analysis: Overlap finding option a) reports overlap percentage with respect to the total Dataset-A regions and percentage with respect to the other active dataset regions. Overlapping or non-overlapping regions of Dataset-A can be extracted by options b), d) or e). Whereas, option c) or f) can be used to extract overlapping sections of regions common in all or two datasets. The results of overlap analysis type b), c), d), e) and f) can be saved back into the Knowledge Base by the ‘Save to KB’ button, allowing them to go into downstream analysis and independent annotation. Ticking the “Extract both datasets, bp overlap and centre distance between the regions” for options d), e) and f) displays both Dataset-A and Dataset-B regions, bp overlap and distance between the two sets.

This is the main analysis screen where users can analyse active datasets. Six types of analysis are provided: a) calculate percentage overlap of all active datasets, b) extract regions that overlap with all active datasets, c) extract overlapping sections of regions common in all active datasets, d) extract regions that overlap between two selected datasets, e) extract regions that do not overlap with another selected dataset, f) extract overlapping sections of regions common in two datasets. Analysis can be restricted by chromosome. The options a), b) and c) operate on all active datasets while options d), e) and f) are designed to work on two selected datasets. Ticking the “Extract both datasets, bp overlap and centre distance between the regions” for options d), e) and f) displays both Dataset-A and Dataset-B regions, bp overlap and distance between two sets. The number of base pairs (bp) either in common in two sets (set by a positive number) or separating two sets (set as a negative number) can be specified, as can be the maximum allowed distance between the centres of two compared regions. Overlapping results can be visualized as Venn diagrams or saved to the KB or a tab delimited text file (Figure 3, circled).

**Figure 4:**
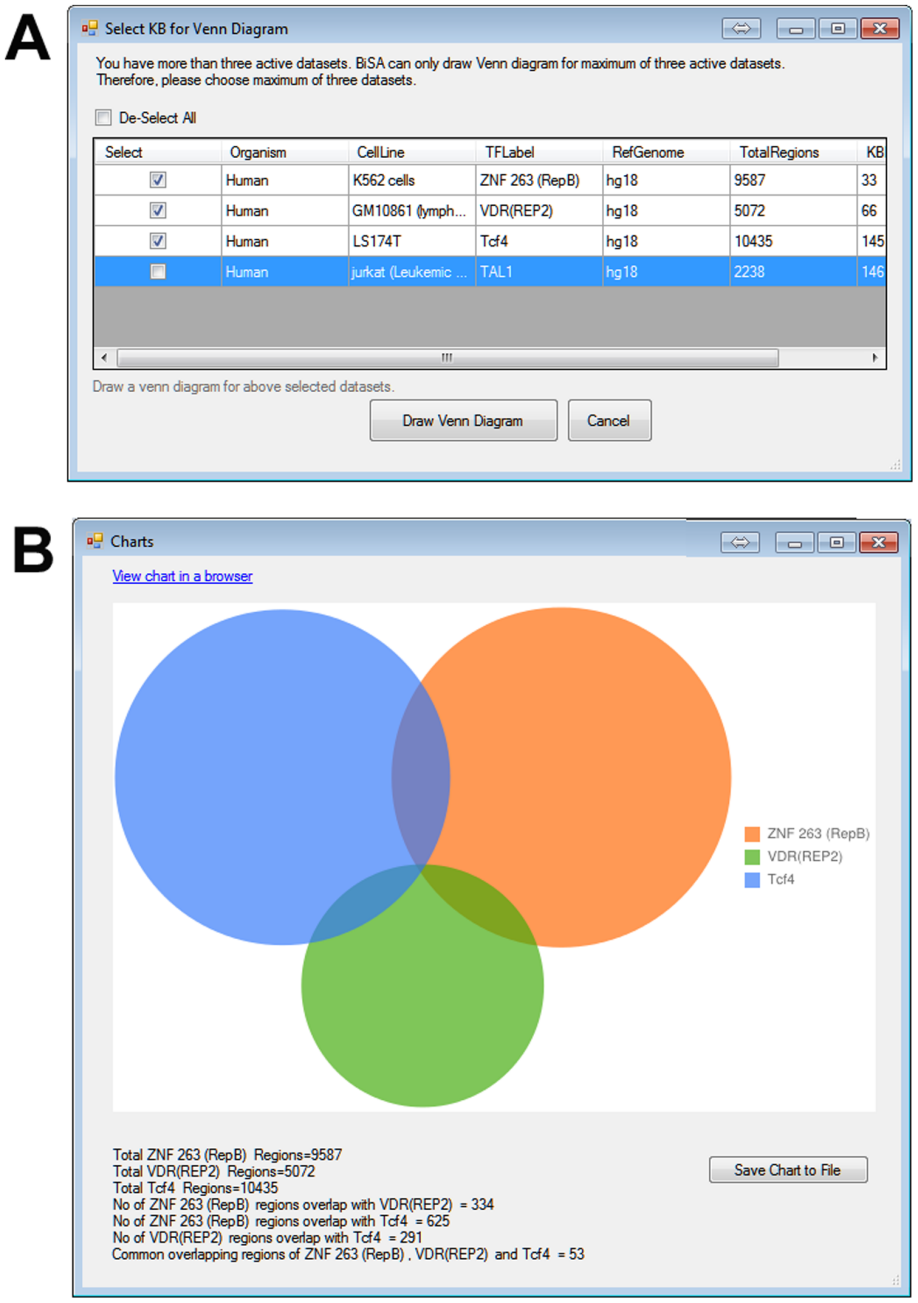
Venn diagrams. BiSA can cross-compare a maximum of three active KB as a Venn diagram. (A) If there are more than three active datasets then a pop-up window appears that allows the investigator to select three datasets to be analysed. (B) Google Charts is used to draw Venn diagrams. The diagram can be saved as a high quality PNG file.

All analyses require setting minimum ‘bp overlaps’, however, specifying maximum distance allowable between two binding peaks or limiting results to a chromosome is optional. A positive value for minimum bp overlap would restrict results for regions that share the specified number of common base pairs. For example, while studying TFs that compete for a specific DNA sequence or finding TFs that form a complex and bind to DNA, the minimum bp overlap can be set to 1 and maximum distance from the centre of two sets should be small, such as 50 bp. To study TFs that potentially bind close to each other but without overlap, a negative value of minimum bp overlap can be assigned to report nearby regions. For example assigning a bp overlap of −100 will report nearby regions separated by up to 100 bases, in this case, a maximum centre distance should be specified. The analysis results section is a data grid that populates the results of the performed analysis (Figure 3, circled). Results can be saved in a tab delimited text format, to allow further analysis in other software. Results can also be sorted by selecting any column heading. The Venn diagram button visualizes overlaps of a maximum of three activated datasets (Figure 3, circled). If there are more than three active datasets, BiSA displays a warning (Figure 4A). Overlap statistics are displayed below the Venn diagram, which can also be saved to a file for later reference or figure preparation (Figure 4B). Overlapping or non-overlapping regions can be saved back to the KB (Figure 3) allowing them to go into downstream analysis and independent annotation.

#### iv) Statistical Significance

**Figure 5:**
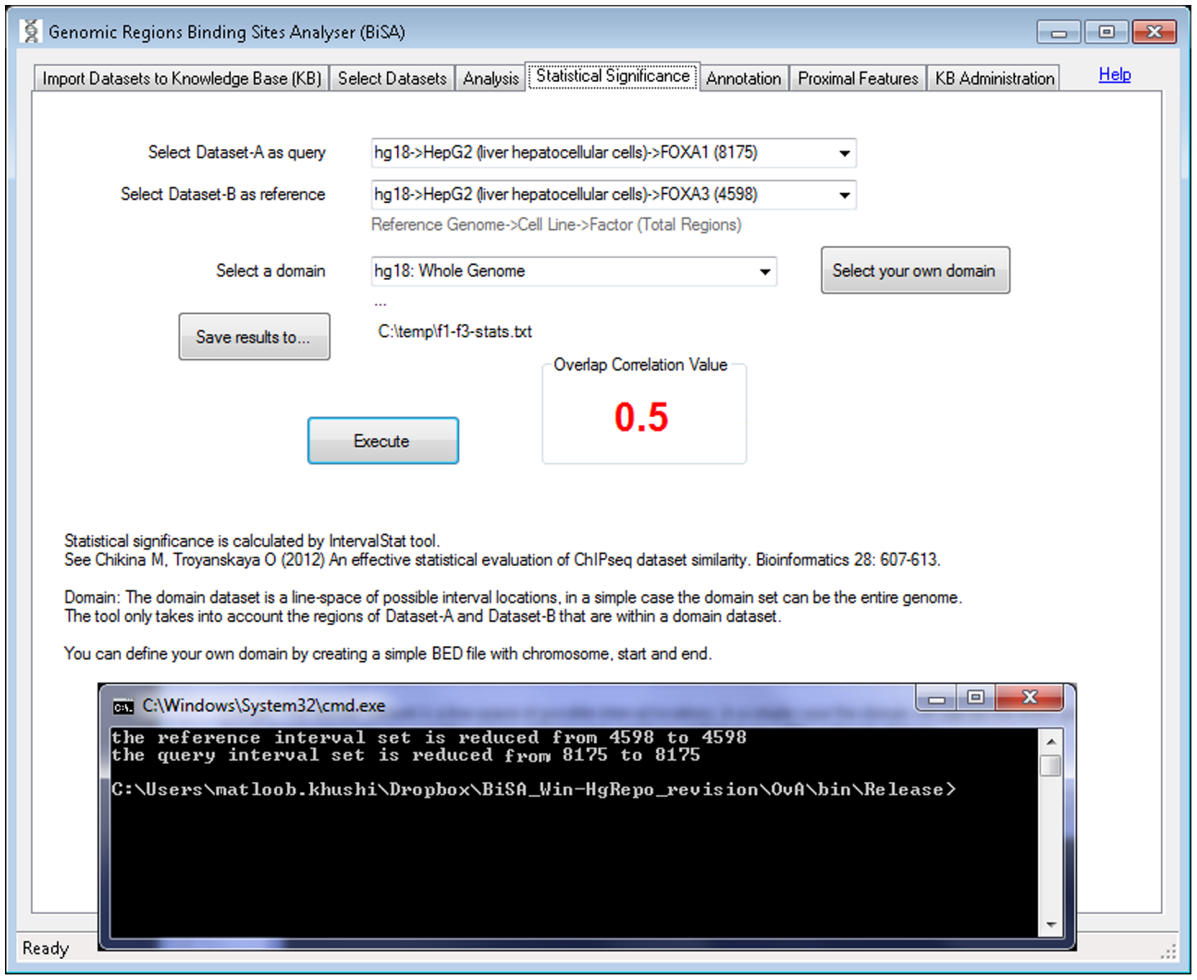
Statistical significance of overlapping regions. The statistical significance tab allows the user to determine the statistical significance of the extent of overlap of two sets of regions. Active datasets are loaded into two dropdown lists and the user selects one dataset as a query and the other one as a reference. Only regions of both datasets that are within the selected domain dataset are included in the calculation. Clicking the Execute button calls up a command-line window and executes the IntervalStat tool. The command-line window stays open to display the messages from the tool. When the terminal window is closed BiSA calculates Overlap Correlation Value of the two datasets.

The number and location of TF binding regions discovered in a ChIP-seq experiment is influenced by experimental design, model used, sequencing depth and analysis approach. Therefore, this information is made available in as much detail as possible in BiSA, so that users can make judgements about the appropriateness of specific dataset comparisons. To determine the level of statistical significance of the degree of overlap in two datasets, the IntervalStats command line algorithm [10] is implemented in a user friendly graphical interface. Active datasets to be compared are selected via two dropdown lists (Figure 5). Users can select one dataset as a query and the other one as a reference. IntervalStats only takes into account the regions that are within a defined domain dataset, representing the total available genomic area for binding. The results are saved as a tab delimited text file with the regions from Dataset-A (query) and Dataset-B (reference), Dataset-A region size, the distance between them and the corresponding numerator and denominator used to calculate the p-value, which is saved as the last column. Once the IntervalStats tool finishes the process and the user closes the terminal window, BiSA calculates and displays an Overlap Correlation Value as described in the Methods section, which reflects the overall significance of overlap of the two datasets.

#### v) Annotation

**Figure 6:**
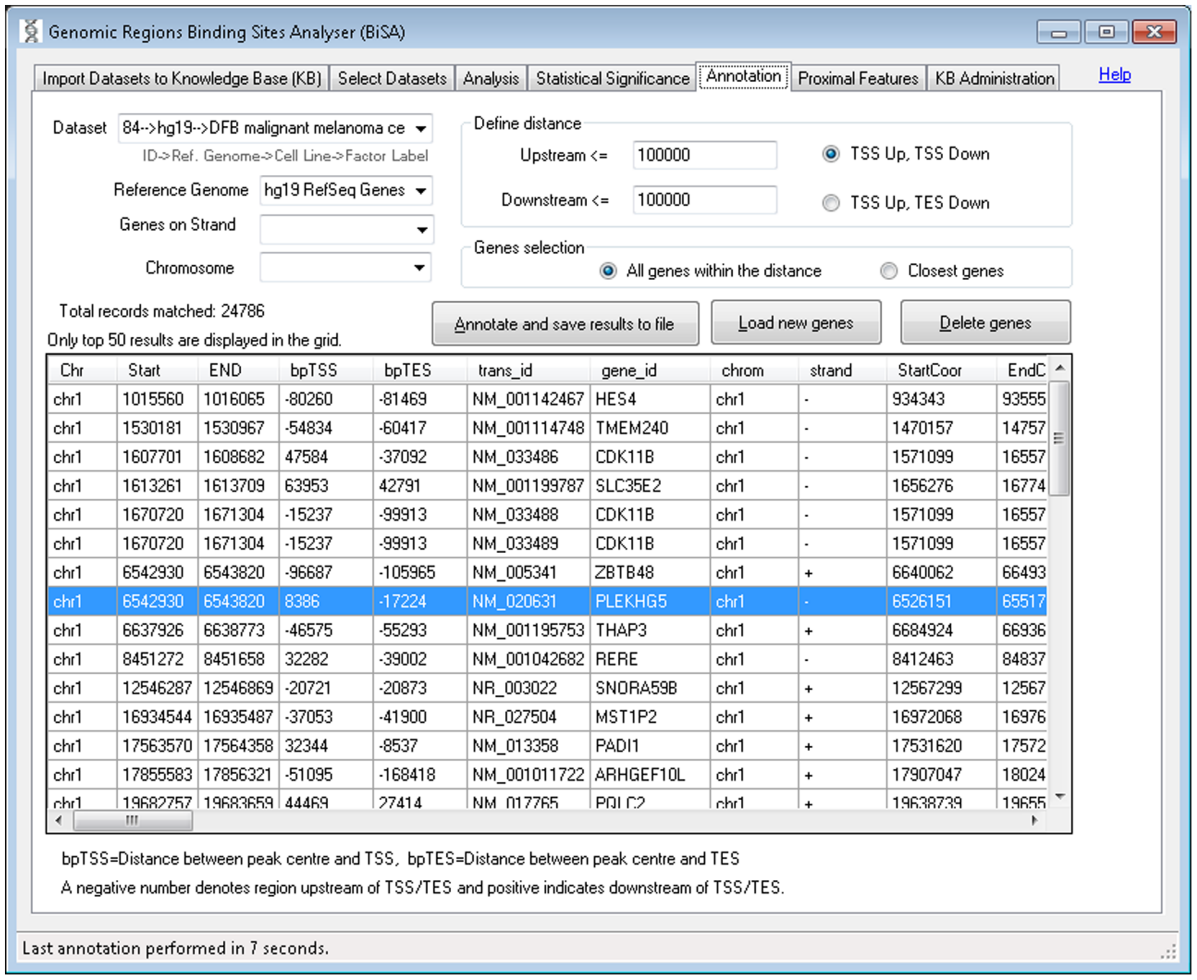
Gene annotation. The annotation tab allows the user to add gene information, from human and mouse reference genome assemblies, taken from the UCSC Genome Browser, to their data. This data can be saved in tab delimited text format for further analysis in other software. Annotation can be limited to a chromosome and strand. Start and End co-ordinate columns for transcript (tx) and cDNA (cds) represent the numerically lower and higher value chromosomal coordinates for genes on both strands. A negative value in the bpTSS or bpTES column indicates that the region is upstream of the annotated TSS or TES respectively. Therefore a region within a gene on the positive strand will have a negative bpTES value and a positive bpTSS value as for the region highlighted. Only the top 50 results are displayed in the grid, however, the full annotated dataset is saved in a tab delimited text file which can be opened in Excel or other spread sheet management software for further analysis. The delete genes button allows the user to delete custom uploaded definitions.

**Figure 7:**
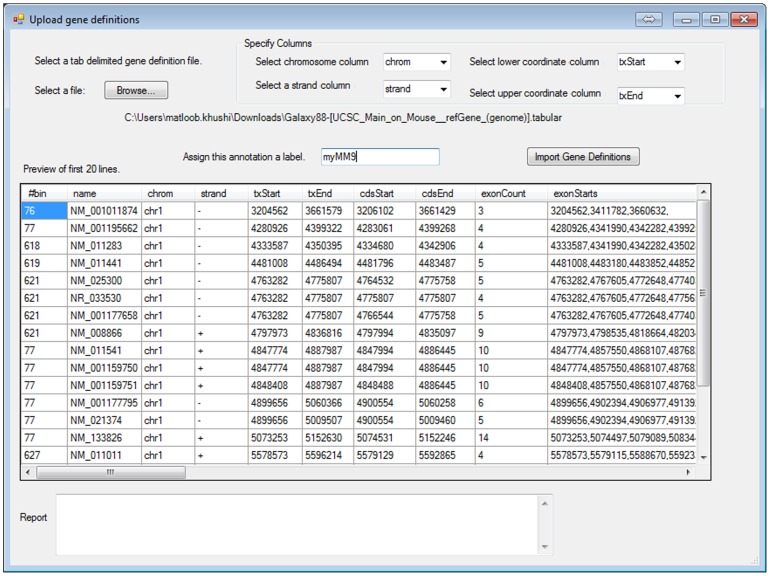
New gene definitions. New gene definitions may be uploaded in the software. The user must specify columns for chromosome, strand, lower and upper coordinates.

The annotation tab (Figure 6) allows the user to add nearby gene information to a selected set of binding regions. Users define maximum distances between binding peak and transcription start and end sites of nearby genes. The nearest gene per region or all genes within the designated number of bp limits will be reported. Selecting “Load new genes” (Figure 6) allows custom gene definitions for additional organisms to be uploaded (Figure 7). The delete genes button allows the user to delete the custom uploaded definitions.

#### vi) Proximal Features

**Figure 8:**
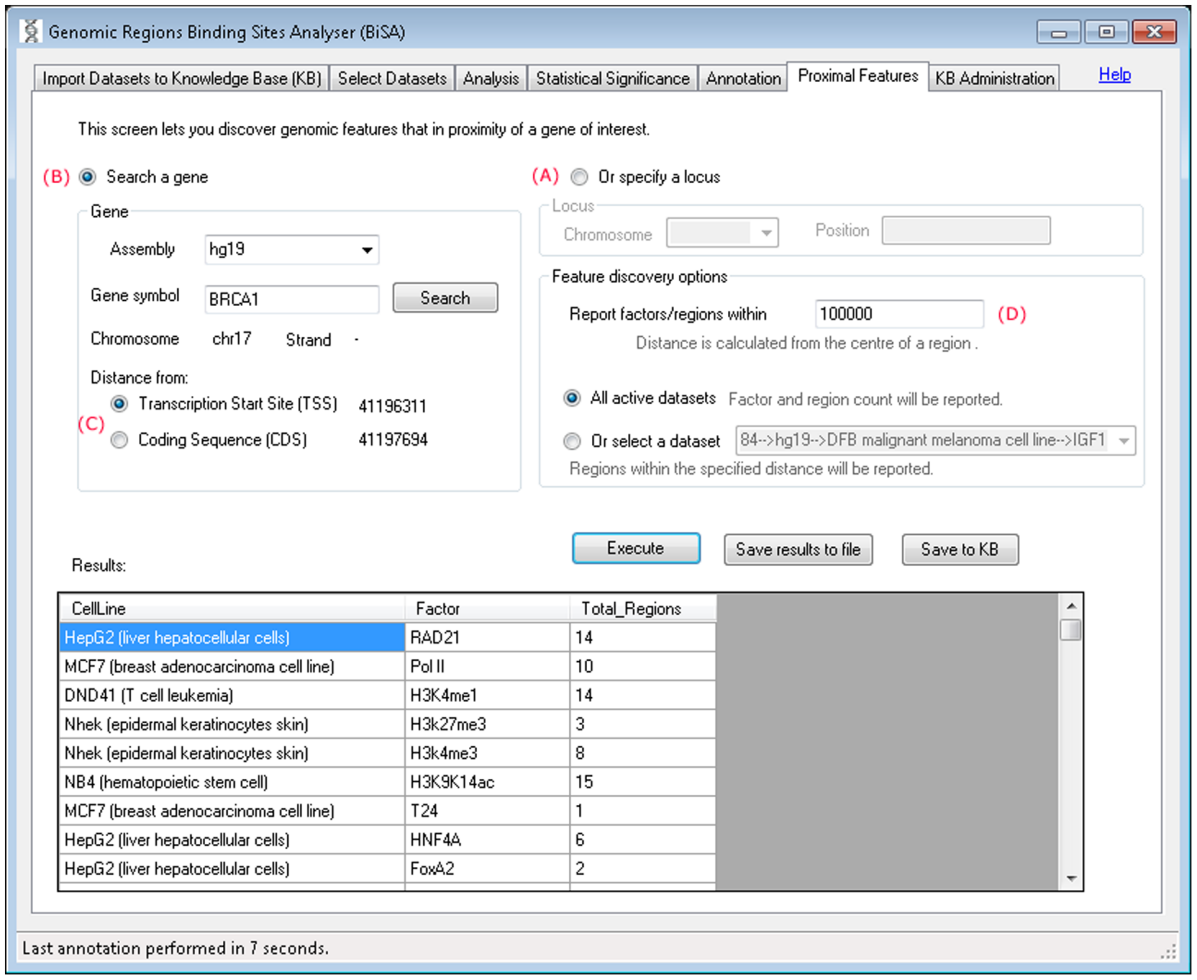
Proximal features. This tab allows users to search for genomic features located in proximity to a specific gene or genomic locus. Searching multiple datasets returns the numbers of binding sites for each factor identified. Selecting a single factor returns detailed binding region information for interactions in proximity to the gene or locus of interest.

This tab lets the investigator discover features that are in proximity of a gene of interest. The nearby genomic features can be discovered by specifying a locus, chromosome and position (Figure 8A) or a gene (Figure 8B). The gene can be searched by specifying an assembly such as hg19 and typing the exact gene symbol or typing the first few letters of the gene name and pressing the Search button which brings up a list of matching genes. Once a gene is selected, its chromosome, strand, TSS and CDS are displayed and the user can select whether the distance should be calculated from the gene TSS or CDS (Figure 8C). The distance between genomic features and the regions is calculated from the centre of the regions and can be set (Figure 8D). Selecting ‘all active datasets’ reports cell line, feature/factor and total regions found within the specified distance. If user selects a single KB dataset then full details of all regions within the specified distance are reported which can then be saved back into the KB. All results can also be saved to a file.

#### vii) Administration

**Figure 9:**
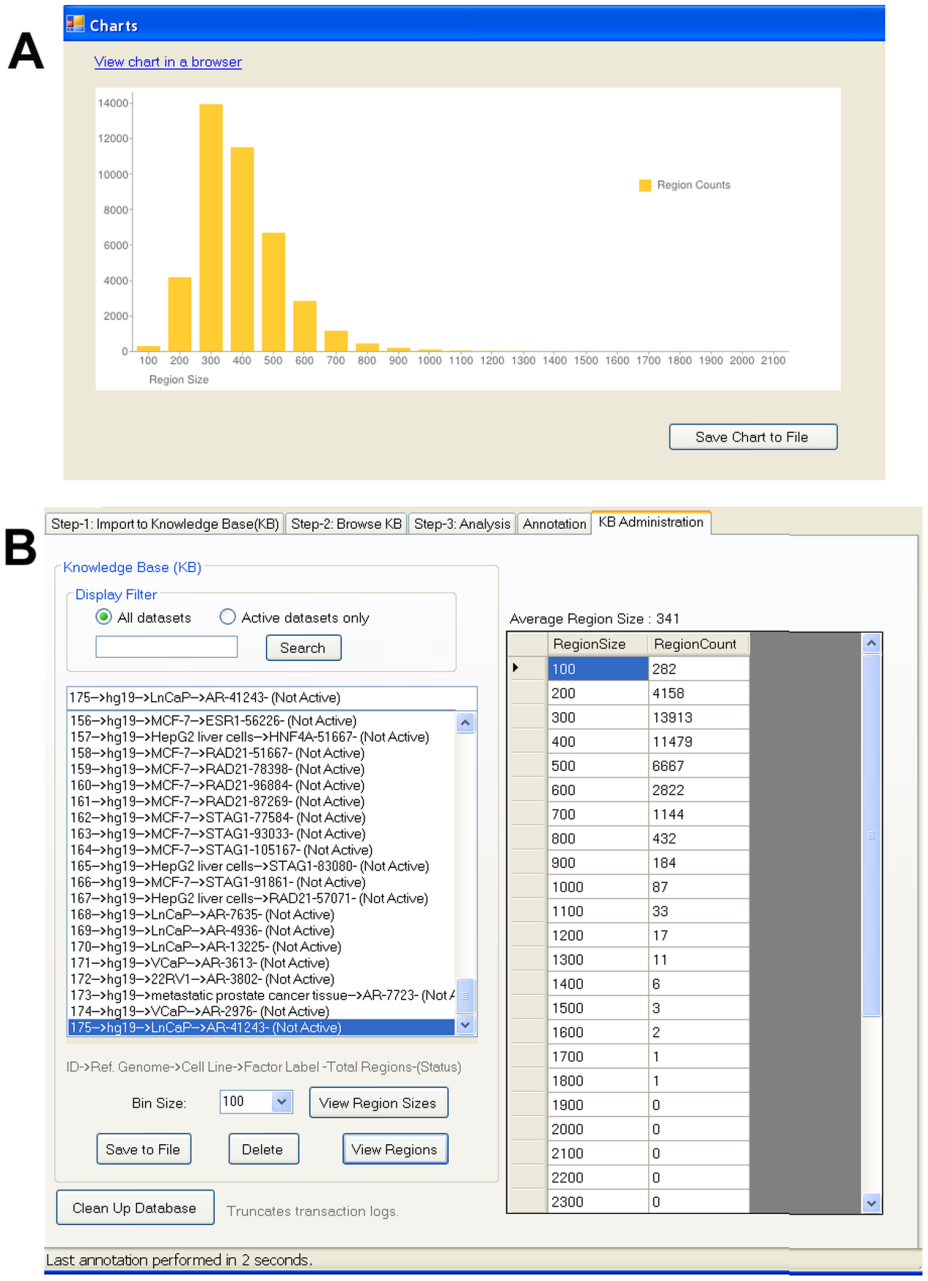
Administration of datasets. From the Administration tab users can delete a dataset, save the data in tab delimited text format, and view the distribution of region sizes over the dataset as a table and histogram.

From the Administration tab (Figure 9) users can delete a dataset, save selected data in a tab delimited format, and view regions or region sizes. The distribution of region sizes over the dataset can also be listed or can be visualised as a histogram (Figure 9A) The Clean Up Database button truncates transaction logs, to avoid an impact on software performance.

#### BiSA Application Example

To present the BiSA utility, we have studied six hg18 datasets available in the KB, transcription factors FoxA1 against FoxA3 [12], CTCF against SA1 [13] and ZNF263 against c-Fos [14]. The forkhead family of pioneer factors (FoxA1, FoxA2 and FoxA3) play important roles in early development to metabolism and homeostasis in adults, and are required for regulation of liver specific genes [12], [15], [16]. Their DNA-binding domains are highly conserved from yeast to mammals, and there is evidence for cooperative function between the family members [12], [16], [17]. FOXA factors are pioneer factors due to their ability to bind condensed chromatin and reposition nucleosomes, allowing the binding of other factors [16]. These TFs work together in complex ways to regulate transcription, therefore, the co-location of binding sites of these factors has been extensively studied in the HepG2 cell line [12], [18]. Here we demonstrate the application of BiSA by investigating the overlap of binding sites for FoxA1 (8175 regions) and FoxA3 (4598 regions) [12] in HepG2 cells. We have also examined the dataset of Schmidt et al. for the overlap between CTCF and the cohesin complex component SA1 which are known to collocate on DNA [13]. In addition we also studied two non-related transcription factors c-Fos (18211 regions) [14] and ZNF263 (4426 regions) [19] in the K562 (erythromyeloblastoid leukemia) cell line.

**Figure 10:**
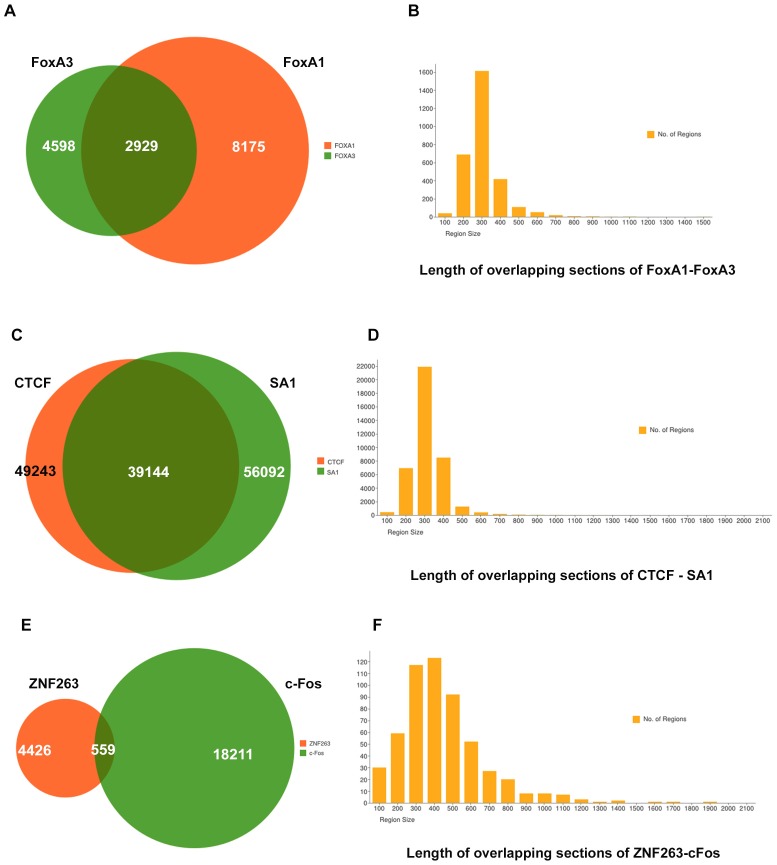
Example study of overlap between FoxA1 and FoxA3, CTCF and SA1, ZNF263 and c-Fos datasets. A) Venn diagram representation of 2,929 overlapping regions in FoxA1 (8,175 regions) and FoxA3 (4,598 regions) datasets. B) Common sections of overlapping regions are saved back into the KB, and for bin size 100 a histogram of the size distribution of region overlaps is drawn. The histogram shows that there are more than 1,600 regions that have at least ∼300 bp in common between the two datasets. C) Overlap of 39,144 regions between CTCF and SA1 datasets. D) Distribution of overlapping sections of CTCF and SA1. E) Overlap of 559 regions between ZNF263 and c-Fos datasets. D) Distribution of overlapping sections of ZNF263 and c-Fos. We also observed that in three comparisons >94% of the overlapping sections are >200 bases long, suggesting that overlapping regions usually share a significant section of the two sets.

BiSA overlap analysis of FoxA1 and FoxA3 with at least 1 bp in common reported 2929 FoxA1 regions. To show this interaction graphically we drew a Venn diagram (Figure 10-A). When we extracted the overlapping common sections of the regions the number increased to 2939 regions which shows that some regions of the two datasets overlap more than one region of the other dataset. We saved the overlapping sections back into the KB. ‘View Region Sizes’ under the Administration tab is used to draw a histogram of region sizes using bin size 100 (Figure 10-B). The histogram, showing the distribution of overlapping region sizes, reveals that ∼99% of overlaps exceed 200 bases and there are more than 1600 regions that have at least 300 bp in common between the two datasets. Similarly the overlap analysis (39,144 common regions) of CTCF (49,243 regions) and SA1 (56,092 regions) is drawn as a Venn diagram and overlapping sections are represented in a histogram (Figure 10-C,D). Similar to the FoxA1-FoxA3 example, the number of common overlapping sections (39586) is greater than the total number of CTCF binding sites due to the fact that a subset of regions overlap multiple regions in the comparison dataset. By contrast, when the unrelated TFs, c-Fos and ZNF263, are compared, just 559 overlaps are detected. A Venn diagram showing the dataset overlap and a histogram summarizing the overlaps are drawn (Figure 10-E,F).

We annotated the common sections of regions to observe their distribution and distance from the nearest TSS by setting criteria of 100K bp up and downstream from TSS and extracted annotations closest to genes. BiSA reported 3656 gene annotations for FoxA1-FoxA3 overlapping sections, 45,508 annotations for CTCF-SA1 sections and 810 annotations for ZNF263-c-Fos sections. The annotation files also contain the distances from the TSSs.

Finally we investigated the statistical significance of overlap for each of the example comparisons. We calculated p-values for all regions in both datasets for each comparison using the hg18 whole genome domain. Selecting FoxA1 as query and FoxA3 as reference returned an overlap correlation value (OCV) of 0.50. By contrast, if FoxA3 was compared as query to FoxA1 as reference, the OCV was increased to 0.72. This provided an average OCV value of 0.61. An average OCV above 0.5 suggests that two datasets significantly overlap, implying that the overlap between FoxA1 and FoxA3 is statistically significant. The high degree of overlap between CTCF and SA1 also returned a significant average OCV at 0.79. By contrast the lower level of overlap seen between ZNF263 and c-Fos was reflected in a non-significant average OCV of 0.21, confirming that the two TFs are not related and do not act on the same DNA regions in general.

## Discussion

BiSA has been designed to meet the challenges of identifying genomic region overlaps in whole genome datasets. BiSA includes an up-to-date database of previously published studies reporting binding sites for different factors and specific histone modifications in a range of conditions and cell types. No tool, to our knowledge, includes such a pre-loaded knowledge base. Initially we have included data generated from human and mouse cells, and expansion to other organisms is planned. BiSA provides a user-friendly interface allowing the user to define and discover overlapping and nearby genomic regions either limited by chromosome or genome-wide. Users can visualize genomic overlap results as Venn diagrams and can save chart images for use in publications. BiSA can identify genes associated with binding regions of interest and also the statistical significance of overlapping regions.

Although the Apple Macintosh Unix and Linux environments are popular in genomic research, Windows based informatics tools also exist [20], [21], [22]. BiSA for Windows exploits the power of today's multi-core personal computers. In comparison to BiSA, most bioinformatics tools are command line, and such tools are not easy to install or to operate by the bench biologist. Galaxy [7] offers a web-based tool ‘Intersect’, however it is limited in functionality. BiSA's Windows GUI is user-friendly for biologists and provides a sequential step-by-step guide through all the options. BiSA provides an easy interface to search and select KB based on organism, factor, cell line, condition, peak caller or first author name. We have also developed a BiSA version for Linux/Mac that runs under Galaxy.

A major strength of BiSA is the comprehensive knowledge base, coupled with tools to analyse overlapping regions, statistical significance of the overlapping regions and ability to annotate and visualize the regions of interest. BiSA's comprehensive KB is not only useful for rapid comparison of users' own results to previously published datasets, but also to inform decisions such as selection of a peak caller programme or in comparing numbers of peaks. The KB suggests that MACS is a most popular peak caller software in ChIP-Seq studies followed by Cisgenome and HOMER, whereas, the MAT algorithm is widely used in ChIP-chip studies. In summary, BiSA is designed for ease of use on a Windows platform, and includes a comprehensive knowledge base of binding site and histone modification datasets. BiSA has the potential to be a useful tool in identifying overlaps in genomic binding regions and histone modifications of common transcription factors.
